# Annotated dataset for retinal fundus image segmentation: vascular arcade and optic nerve head

**DOI:** 10.1186/s13104-026-07957-6

**Published:** 2026-08-07

**Authors:** Bryan Alejandro Figueroa-Garza, Betsaida Lariza López-Covarrubias, Laura Johana González-Zazueta, Christian Xavier Navarro-Cota, Mabel Vázquez-Briseño, Juan Iván Nieto-Hipólito, Gener José Avilés-Rodríguez

**Affiliations:** 1 Escuela de Ciencias de la Salud, Universidad Autónoma de Baja California, Carretera Transpeninsular S/N, Valle Dorado, 22890 Ensenada, BC México; 2 Facultad de Ingeniería, Arquitectura y Diseño, Universidad Autónoma de Baja California, Carretera Transpeninsular 3917, Playitas, 22860 Ensenada, BC México

**Keywords:** Computational ophthalmology, Annotation, Fundus imaging, Deep learning, Artificial intelligence, Vascular arcade mask, Optic nerve mask, Segmentation

## Abstract

**Objectives:**

Digital fundoscopy is an ophthalmological technique used to evaluate the posterior segment of the eye, including the retina and its anatomical structures [1]. This technique acquires fundus images, visually documenting the aforementioned structures. This dataset was generated from a subset of images from the public Asia Pacific Tele-Ophthalmology Society 2019 Blindness Detection (APTOS 2019 BD) dataset [2], these were manually annotated to generate segmentation masks of anatomical fundus structures, specifically the vascular arcade and the optic nerve head. We contribute to the development and evaluation of artificial intelligence models under comparable conditions by generating data to serve as input for segmentation models, thereby supporting the design of more efficient algorithms for the automated identification of ophthalmological structures [3, 4].

**Data description:**

This dataset consists of 500 masks generated from 500 images randomly selected from the APTOS 2019 BD dataset [2]. These are non-homogeneous color fundus images containing global labels for pathological alterations, illumination, as well as varying image resolutions. Image dimensions vary from 474 × 358 pixels to 4288 × 2848 pixels. This dataset provides manual masks for specific ophthalmic structures.

## Objective

Medical image segmentation is fundamental for clinical diagnosis and disease monitoring, particularly in ophthalmology, where detecting vascular structures and the optic nerve head is linked to crucial steps for identifying conditions such as diabetic retinopathy and glaucoma [3]. Digital fundoscopy evaluates the posterior part of the eye [1]. With this technique, it is possible to obtain fundus images, which visually document these structures.

This dataset was compiled from the publicly available Asia Pacific Tele-Ophthalmology Society 2019 Blindness Detection dataset (APTOS 2019 BD) [2], with the purpose of providing segmentation masks derived from its images, so that other researchers can use them to evaluate their models under comparable conditions. This dataset is valuable because it provides manual masks for specific ophthalmic structures: Vascular arcade [5] and optic nerve head [6]. Such masks can be utilized for the parameter tuning of image segmentation models, which can be reused for comparative evaluations (benchmarking) that compare the capability of computational models to identify ocular fundus structures against human performance. It can be utilized to train artificial intelligence models that perform the segmentation of ophthalmic anatomical structures, thereby contributing to the development of more robust and specialized algorithms [3, 4].

This dataset is being released under the Apache 2.0 license, reason being that it is permissive, offering the greatest possible freedom to research groups for reproduction, and allows derivative uses in multiple research contexts (such as industry and academia).

## Data description

The dataset consists of annotation masks from fundus digital images, corresponding to the optic nerve head and vascular arcade [7]. Table 1 presents the overall content of the data presented by the authors: The masks dataset in both standard and visualization focused encoding, annotations in COCO format, a Python 3 script to build the mask images from the annotations, a methodology document, Tables 2 and 3, describing image dimensions and annotated structures, respectively. The dataset consists of 500 masks generated from 500 randomly selected images from the APTOS 2019 BD dataset hosted on Kaggle [2]. The selected color images, like the source dataset, comprise non-homogeneous color fundus images with varying illumination and resolution. Image resolutions range from 474 × 358 to 4288 × 2848 pixels, as shown in Table 2.

**Table 1:** Overview of data files and datasets

Label	Name of data file/data set	File types (file extension)	Data repository and identifier (DOI or accession number)
Data set 1	Masks	.png	Zenodo https://doi.org/10.5281/zenodo.20711325 [7]
Data set 2	Visualized_masks	.png	
Data file 1	Annotations_COCO.json	.json	
Data file 2	JSON_COCO_to_PNG_masks.py	.py	
Data file 3	Table 2. Dimensions and the number of images corresponding to each resolution.	.xls	
Data file 4	Table 3. Description of the structures and their visualization	.xls	
Data file 5	Methodology	.txt	

**Table 2:** Dimensions and the number of images corresponding to each resolution

Resolution (px)	Number of images	Resolution (px) (continued)	Number of images (continued)
1050x1050	108	4288x2848	9
2416x1736	87	1844x1226	7
2588x1958	73	640x480	6
3216x2136	60	2144x1424	4
2048x1536	51	2896x1944	3
819x614	48	1476x1117	3
3388x2588	24	474x358	1
1504x1000	16		

Annotations were performed using the open-source Computer Vision Annotation Tool (CVAT) [8]. CVAT facilitated the simultaneous annotation of two key anatomical structures, vascular arcade and optic nerve head, whose characteristics are detailed in Table 3 across the 500 selected original images. The “brush” tool was used because it was the only feature capable of accurately tracing the irregular shapes of both structures. A Logitech MX Vertical computer mouse [9], configured to 1600 Dots Per Inch (DPI) was used for annotation.

Upon completion of the annotations, the mask dataset was exported from the platform following the Common Objects in Context (COCO) data structure format [10], version 1.0 (standard with Run-Length Encoding segmentation), encapsulated within JavaScript Object Notation (JSON) files. From these files, PNG format masks were generated, wherein each pixel is labeled with an integer value from 0 to 2, depending on its corresponding class: 0 for the background, 1 for the vascular arcade, and 2 for the optic nerve head. A Python 3 script converted the JSON annotations into PNG masks. This script iterated through all the images referenced in the JSON file and merged the category-specific annotations into a single final mask per image. The process used *NumPy*, *OpenCV*, *pycocotools*, and *tqdm* libraries to decode the COCO-formatted annotations, generate the pixel-encoded masks, and save them in PNG format. Additionally, the *os* library was used for path management within the Ubuntu GNU/Linux 22.04.4 LTS operating system. The generated masks preserve the exact resolution and correspondence with the original images from the APTOS 2019 BD dataset [2]. To differentiate them from the original images, the suffix “mask” was appended to the original file name of each mask, thereby maintaining a direct relationship between each image and its respective mask. The script used is available in the repository.

To provide flexibility, the pixel values and output image format can be modified according to the needs of the research team that decides to use the files by using the JSON file, allowing the encoding to be adapted to their own criteria.

**Table 3:** Description of the structures and their visualization

Structure name	Description	Visualization
Vascular arcade	The set of anatomical entities (ophthalmic veins and arteries) visible during funduscopy.	
Optic nerve head	The structure of the nervous system responsible for transmitting visual stimuli from the retina to the brain.	

## Limitations

Although the eye contains diverse anatomical structures, this dataset focuses on two of the most representative regions: the vascular arcade and the optic nerve head. On the other hand, the sample size of 500 annotated images may restrict the generalization capability of segmentation models trained exclusively with this dataset. Furthermore, the manual annotations, although performed with standardized tools, may exhibit variability due to human bias and the diversity of the APTOS 2019 BD dataset [2], whose varying levels of illumination, contrast, and noise make the precise delineation of the structures of interest difficult in some cases.

## Data Availability

The data described in this data note can be freely and openly accessed on Zenodo under https://doi.org/10.5281/zenodo.20711325.

## Abbreviations

APTOS: Asia Pacific Tele-Ophthalmology Society
COCO: Common Objects in Context
CVAT: Computer Vision Annotation Tool
JSON: JavaScript Object Notation
PNG: Portable network graphics

## References

[1] Bernardes R, Serranho P, Lobo C. Digital Ocular Fundus Imaging: A Review. Ophthalmologica. 2011;226(4):161–81. 10.1159/000329597. 21952522

[2] APTOS 2019 Blindness Detection. https://kaggle.com/aptos2019-blindness-detection. Accessed 18 Oct 2025.

[3] Fu H, Cheng J, Xu Y, Wong DWK, Liu J, Cao X. Joint Optic Disc and Cup Segmentation Based on Multi-Label Deep Network and Polar Transformation. IEEE Trans Med Imaging. 2018;37(7):1597–605. 10.1109/TMI.2018.2791488. 29969410

[4] Ronneberger O, Fischer P, Brox T. U-Net: Convolutional Networks for Biomedical Image Segmentation. In: Navab N, Hornegger J, Wells WM, Frangi AF, editors. Medical Image Computing and Computer-Assisted Intervention – MICCAI 2015. Lecture Notes in Computer Science. Cham: Springer International Publishing; 2015. pp. 234–41. 10.1007/978-3-319-24574-4_28.

[5] Schneiderman H. The Funduscopic Examination. In: Walker HK, Hall WD, Hurst JW, editors. Clinical Methods: The History, Physical, and Laboratory Examinations. 3rd ed. Boston: Butterworths; 1990. 21250045

[6] Smith AM, Czyz CN, Neuroanatomy. In: StatPearls, editor. Cranial Nerve 2 (Optic). Treasure Island (FL): StatPearls Publishing; 2025. 29939684

[7] Figueroa Garza BA, Lopez Covarrubias BL, Gonzalez Zazueta LJ, Vazquez M, Navarro-Cota CX, NIETO HIPOLITO JI., Annotated dataset for retinal fundus image segmentation: Vascular arcade and optic nerve head. Zenodo; 2026. 10.5281/zenodo.20711325

[8] CVAT.ai Corporation. Computer Vision Annotation Tool (CVAT) [Python]. 2023. https://github.com/cvat-ai/cvat. Accessed 18 Oct 2025.

[9] Logitech. Mouse inalámbrico ergonómico Logitech MX Vertical. https://www.logitech.com/es-ar/shop/p/mx-vertical-ergonomic-mouse. Accessed 18 Oct 2025.

[10] The COCO dataset format - Rekognition. https://docs.aws.amazon.com/rekognition/latest/customlabels-dg/md-coco-overview.html. Accessed 21 Nov 2025.

[11] WMA - The World Medical Association-WMA Declaration of Helsinki – Ethical Principles for Medical Research. Involving Human Participants. https://www.wma.net/policies-post/wma-declaration-of-helsinki/. Accessed 21 Nov 2025. 10.1001/jama.2024.21972 39425955

